# White masculinity and the radical right in Europe: an intersectional analytical framework

**DOI:** 10.3389/fsoc.2025.1611191

**Published:** 2025-07-17

**Authors:** Anna Guildea

**Affiliations:** Department of Political Science and Sociology, Scuola Normale Superiore, Florence, Italy

**Keywords:** radical right, masculinity, whiteness, intersectionality, gender, race

## Abstract

This paper develops an intersectional analytical framework to examine the radical right in Europe, focusing on how white masculinity shapes the identity, ideology, and power relations of the party family and its support. Concepts pertaining to privilege, status threat, and appeals to victimhood thread these analytical levels together, linking the micro-functional behaviours and attitudes of men to more macro-sociological concepts such as hegemonic masculinity and the relationship between masculinity, technology, and capitalism. Building on “superordinate intersectionality,” this paper interrogates several overstretched concepts prevalent in radical right scholarship and critiques the discipline's persistent blind spots, particularly its failure to adequately theorise race and gender. By foregrounding white masculinity in its conceptual and analytical endeavour, this paper offers new frames for understanding the radical right.

## 1 Introduction

There is now a radical right presence altering established patterns of party competition in almost every Western democracy (Inglehart and Norris, 2016). While the party family has been most prevalently distinguished by the ideological feature of “nativism” (Mudde, 2007), it is also demarcated by a salient gender conservatism, used as a primary field to dispute power and establish antagonisms (Cabezas, 2022). In their work on gender and extremism, Stenger and True (2024) argue that the field lacks theoretically informed conceptualisations of the various levels and mechanisms through which gender promotes, reinforces, and counters the phenomenon. This paper will borrow heavily from their framework, widening its intersectional scope to investigate how power relations attached to whiteness and masculinity mutually shape one another in the context of the radical right.

Following discussion on the radical right as a concept, and the potential contention in the applicability of intersectionality in studying it, this paper applies the tripartite framework adapted from Stenger and True (2024) which moves across the analytical levels of identity, ideology, and power relations. At the level of identity, I examine the representation of white men in radical right electorates and party membership, and how gender, race, and class intersect in shaping radical right subjectivities. At the level of ideology, I critique the concept of “nativism” for its failure to account for how race and gender operate co-constitutively in how we define and distinguish the radical right as a political entity. Finally, at the level of power relations, I interrogate the concept of hegemonic masculinity as it is currently used in the literature, first by analysing how it is often applied incorrectly, and then by engaging with more complex applications that I argue still reproduce analytical binaries that are insufficient, particularly when faced with the “classed” element of radical right support. I return throughout the paper to my central argument: that intersectionality is a necessary method for the analysis of the radical right as a political phenomenon.

## 2 The radical right as a concept

Scholars have used the terms “radical,” “extreme” and “far” in varying ways to describe the right end of the political spectrum—with the umbrella term of the “global far right” referring to a range of formations including political parties, movements, networks, subcultures, and paramilitary groups (Miller-Idriss and Pilkington, 2017; Miller-Idriss, 2020). According to Pirro (2023), the far right sets itself apart from the moderate right via more “radically exclusionary” stances. Mudde (2007) raises the problem of relativism in defining what is “radical,” opting to frame it as opposition to some of the key features of liberal democracy, such as political pluralism, and constitutional protection of minorities. Within the far right, the “extreme right” is anti-democratic, while the “radical right” is illiberal-democratic. This paper focuses on what Pirro (2023) refers to as the “institutional frontstage” of the far right—that is, the “radical right” regarding the party family, with particular focus on its electorate.

Seminal research has frequently considered populism a defining characteristic of radical right parties (Betz, 1994; Taggart, 1996; Mudde, 2007). However, while the term is meaningful to describe certain parties, it does not denote or identify a separate party family (Carter, 2017). I forego the label of “populism” to achieve greater extension—not all radical right actors are necessarily populist (Rydgren, 2018; Pirro, 2023)—while potentially reducing intension, to provide a framework that can be applied to the radical right in the wider European context.

While the far-right is undeniably a global phenomenon, extending the analysis of this paper beyond Europe would require careful recalibration of its core concepts. For example, Borges and Zanotti (2024) highlight key divergences between far-right formations in Europe and Latin America, contending the applicability of the concept of “nativism” in the latter. They cite that anti-immigration has not been salient in public debates in the region (Zanotti and Roberts, 2021), and that existing migration patterns are difficult to frame in relation to “threat” to the “cultural homogeneity” of the state (Kestler, 2022). In decolonial contexts, the analysis of central categories in this paper—especially whiteness—requires more thorough consideration, operating in related though fundamentally altered ways within and outside of the imperial core of Europe.

This paper contends that meaningful conceptual distinction of the radical right must confront its deployment of racism and sexism as defining features that differ from other European right-wing formations in form and function. However, this racism and sexism is not exceptional in origin—often framed as formed in “backlash” to social liberalism, mainstream political actors “exoticize” the radical right as a character foil to the liberal establishment, mirrored in scholarship that treats the phenomenon as an aberration in modern Western politics, as opposed to one deeply entangled in its historical foundations. The ideological architecture of the radical right has not emerged in a vacuum: when we critique it, we critique that which is woven into the fabric of European politics at-large, and of Europe, and academia.

## 3 Intersectionality and an analytical framework

This paper applies intersectionality as a method of analysis (Christensen and Jensen, 2014) which treats social categories as mutually constitutive forms of differentiation, though not necessarily operating through identical logics (Collins, 1990). Using intersectionality in this instance might be contentious. Originally developed to centre marginalised identities (McIntosh, 2020), some scholars have hesitated to use it in male- or white-centred studies, wary of appropriating the intellectual labour of Black feminists. Chandrashekar (2020) argues that this “well-intentioned refusal” of intersectionality only serves to weaken and whiten feminist analyses. Parallel tensions bother the study of masculinity from a feminist perspective, whiteness from the tradition of critical race theory, and the study of Europe through a decolonial lens: the fear of returning white European men to centre-stage. I would contend that the motion we take here is not circular, but spiralic. We return to a familiar place from a fundamentally changed vantage point, with different tools and analytic objectives. Our attempts to “re-provincialise” cannot just involve de-centring, but revisitation. As put by Smith (1998) the discipline is so locked-in to masculinist, white, Euro-centric epistemology, these ways of knowing stop us from asking questions about the very identities that lie behind normative assumptions—that probing the unmarked “in a way that makes it appear that the existing social order could be other than it is, is a full, frontal attack on centuries of domination and privilege,” (p. 65).

To this end, the concept of “superordinate intersectionality,” introduced by Leek and Kimmel (2014) and applied to radical right studies by Norocel (2024), is especially useful. While admittedly under-theorised, this form of “intersectionality from above” (Norocel et al., 2020) focuses on how systems of dominance are maintained through axes such as whiteness, masculinity, and heterosexuality. I believe the elaboration on “traditional” intersectionality is important here for several reasons: in an ethical commitment to avoid appropriating the original political-epistemological project of intersectionality, for one. Further, to avoid simplification of intersectionality as a method in treating it as if it can be applied, with its ontological roots in the study of marginalisation, as-is but in reverse. To critically interrogate normative social categories, that which is normative cannot simply be treated as the conceptual mirror image of that which is Othered. We can attempt to implement a deliberate epistemic shift in perspective—to confront dominance and normativity as complex and relational constructs, to trouble the category of the “universal subject.”

I integrate “superordinate intersectionality” within the framework adapted from Stenger and True's (2024) study of gender and extremism. Their framework identifies three analytical levels through which gender promotes, reinforces, and/or counters extremism: identity, which explores who is targeted by or drawn to extremist groups; ideology, which addresses the role ideas about gender play in shaping political ideologies; and power relations, which refers to the hierarchical order of femininities and masculinities that structure the environments in which extremism takes root. The structure of this framework follows outlines provided by scholars such as Kreisky (2014) who argues that any analysis of male power must include analytical levels that distinguish between individual men or men as a social group, the social and political constructions of different masculinities, and the social production of hegemonic masculinity. Similarly, Harding (1983) stresses three interrelated aspects of gender: a structure of personal identity; a way of organising social relations; and a fundamental category through which meaning is ascribed to everything.

Embedding superordinate intersectionality within this framework extends its utility by foregrounding race and class alongside gender as co-constitutive systems of dominance. In turn, the framework offers superordinate intersectionality a more defined set of analytical levels. Together, both approaches offer a mutually reinforcing lens that better captures the complexity of the radical right's appeal, operations, and embeddedness in broader social orders.

## 4 Identity

Connell (2021) characterises identity in terms of how people define themselves in relation and difference to others, and as shaped by historically contingent social conditions. Identity bridges the individual to the collective—as Jardina (2019) notes, it reflects an internalised attachment to a group, serving as a cognitive structure through which individuals experience and act in the social world.

In the following, I consider who is represented by the radical right—a disproportionately white and male political entity—along with the conceptual limitations of terms such as “men's parties” and “masculinity”. I then turn to socialisation and social status, exploring how masculinity, whiteness, and class intersect to inform political behaviour, specifically through perceptions of threat to social dominance.

### 4.1 Representation—“men's parties”

While there is considerable variation across the radical right in Europe, one consistent pattern observed has been its disproportionate maleness compared to other party families (Lubbers et al., 2002; Norris, 2005; Lucassen and Lubbers, 2012; Harteveld et al., 2015; Immerzeel et al., 2015). “Masculinity” has become a heavyweight concept in radical right scholarship: these are “Männerparteien” or “men's parties” (Betz, 1994), led by figures who embody “masculine character” (Meret and Siim, 2013), who project “masculine” politics, discourse, and values (Spierings et al., 2015; Sauer, 2020).

This framing raises several conceptual issues. Most broadly, in terms of representation, almost all forms of public politics are “men's politics” (Connell, 2005). Hearn (2024) notes that while the constellation of “masculinity”—whatever it is—is pervasive in authoritarian, ethno-nationalist, and militaristic politics, so too is it in democratic, socialist, and various activist politics. Second, while male overrepresentation is highlighted as a defining feature of the radical right, a similar overrepresentation of white individuals goes largely unremarked upon, despite research that has identified the electorate as “ethnically homogenous,” and overwhelmingly “native-born” (Betz, 1994; Norris, 2005; Rydgren, 2013). It could be argued that, as with “masculinity,” describing a party as white does not have much discriminating power in the European context. More critically, the lack of direct engagement with whiteness reflects broader European resistance to race as a relevant category (Boulila, 2019; Goldberg, 2001; Lentin, 2008), institutionalised in academia with scholarship relying on neo-racial (Sharma, 2015) euphemisms like “cultural groups” or “minorities” (Lentin, 2017). Avoiding the term “whiteness” obscures that the politics of the radical right directly enables institutional and physical harm, neglect, violence, and the death of racialised men. “Männerparteien”? Which men? Who counts as “men”?

A third issue concerns the growing “feminisation” of the radical right (Scrinzi, 2017). In the electorate, male overrepresentation has varied temporally and geographically (Spierings and Zaslove, 2017), and in cases such as France, is considered to have disappeared (Mayer, 2015). In terms of party membership, Sauer (2020) suggests a clear “feminisation strategy” is underway, aimed at widening electoral appeal, though women involved in this strategy still struggle with “masculine party structures.” Connell (2005) reminds us that such gender integration still occurs “in the context of patriarchal institutions where ‘male is the norm,' or the masculine is authoritative,” (p. 231). It should be noted here that while women in the radical right certainly struggle with masculine structures, they gain moral legitimacy and symbolic capital as defenders of nation, tradition, and racial purity. White femininity should be understood within this framework as both subordinated and privileged, imbued with relational forms of power and agency, benefitting from its embeddedness in racial hierarchies that these parties aim to institutionalise.

Nonetheless, we are left in the conceptually tricky area where on the one hand, the overrepresentation of men does not necessarily distinguish a political entity as “masculine,” while on the other, the increasing representation of women should not disqualify this notion either. The challenge for scholars in the field is to articulate this “masculinity” more clearly, and to distinguish it from that which is present in other political formations. The concept as it is currently used suffers from Sartori's (1970) “stretching,” possessing vast extension at the meaningful loss of intension—both within political sociology at-large, and in radical right scholarship. If “masculine” is the authoritative norm, describing any political entity with the concept does not offer much by way of analytic utility. In radical right studies, as will be illuminated throughout this paper, “masculinity” is instrumentalised at multiple analytical levels simultaneously, operationalised in plural, often confusing ways: to convey numbers of “men,” as a “personality characteristic” one can possess more or less of, as a web of symbolic meanings, as a characterising aspect of ideology, and as a material-discursive entity. Connell (2005) argues that “masculinity” is not a coherent object of knowledge, but a positionality within a broader system of “gender relations” that does not exist, except in relation to femininity and other masculinities. Likewise, the racialised aspects of the radical right transcend overrepresentation of white people: Ahmed (2007) describes whiteness as something “real,” material, and lived, but also as a “cultural entity” of power that works through a collection of other hegemonic norms that empower white supremacy (McIntosh, 2020).

A final note on the level of representation: extensive scholarship has linked the radical right to particular enclaves of social class. For example, Oesch and Rennwald (2018) identify the stronghold of the radical right among “service” and “production workers” across western and northern European countries from 2002 to 2014. Similarly, Kurer (2020) identifies salient sources of radical right support in the United Kingdom, Germany, and Switzerland as emanating from occupations characterised by their highly “routinized” task content. Much literature points to altering social stratification in post-industrial societies as giving rise to new frustrations among those who consider themselves to be “losing out.” Damhuis (2020) challenges the idea of a socio-structurally homogeneous electorate, identifying multiple subgroups that compose this constituency. He argues that the political behaviour of these groups emerges not from one analytically distinct variable, but configurations of interacting factors. Broader work on class or “cleavage theory” has repeatedly revealed the weak explanatory power of “objective” economic variables on increased radical right support (Bornschier and Kriesi, 2013; Margalit, 2019; Gidron and Mijs, 2019). Instead, this literature relies on explanations pertaining to subjective experiences of economic decline or relative status loss (Rydgren, 2013; Kurer, 2020; Kurer and Van Staalduinen, 2022).

Such “class-only” approaches appear to rationalise the racism and sexism of specific constituencies as side-effects of “economic frustrations” —frustrations that have not always been empirically substantiated (Du Bois, 1935; Ciccolini, 2025). While occasionally mentioning the overrepresentation of men in the occupational/classed groups that emerge as relevant, gender is typically reduced to a binary control variable in these studies, with race scarcely addressed at all. To examine the changing organisation of work in Europe without acknowledging occupation as a historically key site for the construction of white European masculinity (Cockburn, 1991; Wight, 1994; Walkerdine and Jimenez, 2012; McIvor, 2013) is a critical oversight. Capitalist accumulation has always relied on gendered racialised hierarchies – which must underpin the very notion of “status loss” that has become so central to narratives of radical right support. Nonetheless, Zalloua (2024) outlines how intersectional perspectives on the resentment of the working class complicates whiteness as an exclusionary site of power: “it is being devoured by global capitalism, so the necessary distinction between who is dominated (the genuine victim) and who imagines themselves to be (the phantasmatic victim) is not static, but demands perpetual revision and supplement,” (p. 118).

The above underscores why approaches focusing only on class, gender, or race, conceptually isolate themselves from accounting for the “identity” of the radical right, and why intersectionality is essential to analyse how these categories interlock to structure *who* the radical right is composed of. While remaining at the micro-level of identity, the following section will trace how an intersectional lens forces us to reckon with how white, masculine, and class “aggrievement” cohere in the making of radical right subjectivities.

### 4.2 Subjectivities—socialisation and social status

The previous section analyses the identity of the radical right at the low level of abstraction (Sartori, 1970) of descriptive socio-demographic characteristics. In this section, I move upward on this ladder to interrogate how masculinity, whiteness and class intersect in shaping not just who supports these parties, but why and how they respond to perceived social change.

Several studies have examined the role of gender socialisation, “the process by which individuals learn the norms, roles, and behaviours expected of them by society based on their gender,” (Stockard, 2006) on radical right support. Coffé et al. (2023) reveal that increased “masculinity” is linked to radical right support—but only among men, who also score higher on this measure of masculinity compared to women. Their findings align with social psychology research that has revealed “more masculine” men to be more defensive than both “less masculine” men and “masculine” women (Hershey and Sullivan, 1977), and that men who fear the failure of meeting the expectations set by masculinity are more likely to support aggressive policies (Willer et al., 2013). As such, the mechanism of “gendered personality” on political behaviour only appears to operate between men, and only in relation to their “masculinity.” Masculinity is conceptualised in such research as a set of personality traits that an individual can possess “more” or “less” of and does not emerge as a sufficient or necessary condition in explaining radical right support.

Across her work, Connell (1985, 1987, 2005, 2021) has critiqued these “gendered personality” or “sex role” models. She argues that they overstate the extent to which enactments like political behaviour are defined by the dichotomy of masculine/feminine, as opposed to power and social relations. Instead of framing political behaviour as a product of “how much” of “a gender” one has, we might consider that in the above research, the nature of the mechanism at-play has more to do with sensitivity to status threat mentioned previously in class-centred research. This mechanism remains gendered, but is also inherently tied to race and class, which gender socialisation research isolates itself from accounting for. For example, Ahmed (2007) points to whiteness as “an orientation that puts certain things in reach, styles, capacities, aspirations, techniques, habits” (p. 154) and must invariably shape what we feel entitled to, and how we are prone to reacting when those expectations go unmet.

“Social status” is a concept derived from Weber (1922), who defined it as a group's relative esteem in society based on shared norms surrounding worth. Breyer (2023) expands on this by showing that status operates both structurally, through hierarchies based on impressive levels of societal consensus, and individually, as a person's “subjective social status” (Gidron and Hall, 2017). People's political behaviour can be shaped by how they perceive changes in status hierarchies, depending on if these shifts are acknowledged in a basic sense, in that they “are part of a coherent narrative through which people see the world,” (Breyer, 2023: 12). Breyer also uses the concept of “status determinant” which varies across social groups and shapes both static and shifting perceptions of status. She distinguishes between economic and cultural status, the latter of which is based on recognition related to gender, race, class, and their intersections. She applies these status determinants to minority groups perceived to be making “status gains” in society, investigating how political camps differentiated along liberal and authoritarian lines perceive these gains. It is also crucial, I would argue, that we apply these “status determinants” to the group whose perception is being tested—taking what we know “representationally” about the radical right to extrapolate that particular identities are not only more likely to perceive status threat, but also to react to it in politically distinctive ways.

“Gendered personality” research on the radical right cannot surpass descriptive statistics that link specific operationalisations of “masculinity” among men to party support. However, if we intersect such findings to “class-centric” explanations that rely on perceptions of status threat, we can elaborate on how this “phantasmatic” victimhood, or “aggrieved entitlement” is racialised and gendered (Kimmel, 2010). It is difficult to conceptually distinguish sensitivity to status threat from socialisation: we are socialised into expecting things, and thus into aggrievement when these expectations go unmet. We're socialised into reacting differently, perhaps more violently to disappointed expectations, or to the success of others. However, these cannot be whittled down to individual “personality” characteristics. What we are socialised into feeling entitled to can only follow that which already exists structurally. We must first be in possession of status to fear its loss, and we must surely be in possession of power to act on those fears so effectively—to contribute to the fastest growing party family in Europe.

In moving upward on Sartori's ladder of abstraction, we connect concrete socio-demographic observations on identity to broader structures of power and entitlement. By articulating masculinity and whiteness as structurally privileged positions, as well as static identity markers, sensitivity to status threat can be understood as an expression of social privilege. The political reactions arising from such perceptions of threat thus reflect defensive mobilisations of historically dominant groups that perceive their dominance as eroding. Framing the rise of the radical right in this way, we are not articulating a historically distinct phenomenon. Kreisky (2014) recalls the genesis of “männerbund” ideology, connected to the development of the bourgeois feminist movement at the end of the nineteenth century that threatened patriarchal power relations, leading to justifications for masculinist strategies of exclusion. Similarly, Schwarzkopf (2014) recalls the Chartist movement in Britain as a method through which men coped with the erosion of male supremacy in the family and the “the material base on which their masculine prerogatives rested,” (p. 27) brought about by the industrial revolution.

## 5 Ideology

In moving from the level of “identity” to that of “ideology,” we shift focus from who radical right actors and supporters are, to the analysis of units of political thought that can be linked to behaviour (Mullins, 1972). I use the concept of ideology as a patterned configuration of ideas and meanings that support or contest political arrangements and enable purposive action (Freeden, 1998). As will be discussed, ideologies are not strictly rational, often containing internal contradictions, remaining powerful nonetheless as they offer intuitive frameworks that bind people to particular visions of society and identity. As such, ideology is a constitutive force, not only in relation to political action, but in the formation of subjectivities (Van Dijk, 2006).

To Haslanger (2017), the purpose of epistemic critique of ideology is to reveal distortion, to uncover the ways in which it is oppressive. This is a little on-the-nose in relation to the radical right: critiquing what is “oppressive” about the ideology of the phenomenon qua social justice reads somewhat reductively. The purpose of this section as such is not to provide a descriptive account of how ideology of the radical right oppresses particular groups. Rather, it aims to interrogate the “unmarked” normative categories that structure both subjectivities and institutional arrangements alike. This points to ironic blind spots at the heart of radical right scholarship: despite its focus in analysing the most overtly racist and sexist political formations operating today, the field frequently fails to meaningfully engage with whiteness, masculinity, and their crucial intersection as structuring categories. I argue in the following that race fundamentally underpins the concept of “nativism,” the ideological construct routinely used to define the radical right as a party family in Europe. From here, I highlight that gender is not merely an adjacent axis of analysis but is deeply intertwined with the racialised imaginaries and functions of nativism.

### 5.1 Nativism and race

One of the least-contested aspects of radical right scholarship is that which distinguishes the phenomenon via the concept of “nativism.” Mudde (2007) defines it as an “ideology which holds that states should be inhabited exclusively by members of the native group (“the nation”) and the non-native elements (persons and ideas) are fundamentally threatening to the homogenous nation-state,” (p. 19). Mudde further declares that the basis for defining what is (non) “native” can include racist arguments but can also be non-racist. Meanwhile, Carter (2017) views nativism as a possible feature of “right wing extremism” but not a necessary one, distinguishing what she refers to as “extreme right parties” via anti-democratic sentiment. Carter's initial conceptual divergence allows her to produce a typology which includes “neo-liberal populist parties” that are “not xenophobic; not racist,” (p. 58). This group includes the Italian Lega Nord, with reference to whom Carter cites, with no trace of irony, Kitschelt's (1995) argument that before the mid-1990s, former leader Umberto Bossi used “xenophobic anti-immigration slurs,” but that they were “not an expression of a biological or cultural racism so much as new efforts to attack the establishment,” (p. 175).

Whether we buy-into this reasoning, or follow conceptualisations proposed by Mudde, both rest on the flawed assumption that the state can be understood as a non-racial entity, side-stepping critical theory that argues the modern state is not only an actor implicated in racist exclusion, but is racially configured and constituted (Goldberg, 2001; Mulinari and Neergaard, 2017). Andrews (2024) highlights the double standard in European reactions to migrants and refugees from Eastern European contexts as opposed to those from former colonies, arguing that “anti-migrant” attitudes have “always been tied to fears of Black and Brown people,” (p. 126). Norocel et al. (2020) similarly argue that nativism entails a relational process where whiteness acts as the unspoken norm against which Others are measured and defined.

Yuval-Davis (1997) notes the rise of “new racism” that emerged during the Thatcher-era, wherein “culture” and “ethnicity” became euphemisms that replaced biological racism as the primary discourse of the right for essentialising notions of genealogical difference. According to Sharma (2015), this “neo-racism” has not only been a process of replacing terminology but has intensified the politics of anti-immigration itself. Examples of the use of this discourse by radical right parties include the framing of the “cultural threat” of Islam as incompatible with laïcité by the Rassemblent National (Fernando, 2014), or with “folkhemmet” or “cultural sameness” by the Swedish Democrats (Mulinari and Neergaard, 2017). There has also been growth in the use of civilisational discourse premised on “western liberal values” by the Alternative für Deutschland (Forchtner, 2019) and the PVV.

It seems contemporary scholarship in the field has become as much a participant in neo-racial discourse as radical right actors themselves: “native” and “non-native” join the list of terms that allow for the continued circumvention of race as a legitimate, necessary category for the analysis of the radical right, that the “citizen” and the “migrant” are (neo)racialised figures (Sharma, 2015; Balibar, 1991). We thus find ourselves, in analyses of the most overtly racist political entities operating today, arguing for the use of race in our foundational concepts—observing the continued subordination of European knowledge production to European colonialism (Dussel, 2003; Mulinari and Neergaard, 2017).

### 5.2 Nativism and gender

More recent scholarship has emphasised gender as a “meta-language” through which inequalities and power are negotiated by the radical right (Dietze and Roth, 2022). De Lange and Mügge (2015) distinguish between “classic” gender issues of the party family, pertaining to family and motherhood, and “newer” issues related to immigration. Nonetheless, some scholars argue that gender is not part of the radical right's “ideological core” at all (Kitschelt and McGann, 1995; Spierings, 2020), suggesting that gender conservatism is too common across party families to be distinctive to the radical right. There appears to be an analytic inconsistency here: while racist sentiments are found across various political formations, this does not precluded scholars from identifying nativism as an ideological feature to distinguish the radical right. Meanwhile, the wide distribution of gender conservatism excludes gender from this same ideological core.

By implementing intersectionality, we can display that “nativism” is as gendered as it is racialised. A primary example is the practise of “Femonationalism” (Farris, 2012): the convergence of the defence of women's rights with xenophobic aims to represent racialised men as sexually deviant aggressors. Meanwhile, racialised women are portrayed as passive victims of “backwards” religious and cultural practises from which they must be liberated—a continuation of the classic practise of “white men saving brown women from brown men,” (Spivak, 1988: p. 93). A similar mechanism is executed via the practise of “homonationalism” (Puar, 2007). This is put to use by radical right parties, pronouncedly the PVV in the Netherlands (Bracke, 2012) whereby white, cis-gendered, middle-class gay men and lesbian women are imbricated into their objectives in the name of defending aforementioned “western liberal values” to serve xenophobic, and particularly Islamophobic (El-Tayeb, 2011) ends. Adjacent to the parasitic appropriation of feminist rhetoric by these parties, “homonationalism” does not approach critical deconstruction of heteronormativity or destabilisation of the obligatory gender binary, but creates hierarchies within the queer community, favouring those that conform to racial and gendered norms. This ascribing of “pre-modern” models of gender and ideation towards sexual minorities to racialised Others serves simultaneously the xenophobic objectives of the radical right, and the construction of the categories of whiteness and masculinity, as composing the morally advanced and superior “national self” (Scrinzi, 2024).

Another example is the clear intrinsic relation between the attack on “gender ideology” and migration highlighted by Butler (2024), which manifests in parallel framings of transwomen and racialised men as sexually deviant aggressors that threaten “biological” women. Enlarged into phantasms of sexual predators, transwomen and racialised men are framed as exemplifying all that is most dangerous about masculine sexual violence, “the implicit point is that someone who has a penis, or even someone who once had one, will rape, because the penis is the cause of rape,” (Butler, 2024: p. 157). Of course, white cis-ness gets specific men off-the-hook from this line of reasoning. Sexual violence is disavowed as belonging to the culture of the racialised Other, or the deviancy of the trans individual—when white men rape, they are violating a norm, but when those who are Othered do so, they are conforming to one (Srinivasan, 2021). Butler (2024) likens the trans individual proliferating “gender ideology” to the racialised migrant—both are portrayed as inherently abusive, “and both are threatening the nation and Europe itself. Gender and race intertwine as a phantasm that threatens national identity,” (p. 254).

Regarding “classic” gender issues in the domain of the “native” family, radical right parties construct what Scrinzi (2024) refers to as the “family/nation symbolic nexus” via the institutionalisation of reproductive heterosexuality. As discussed by Yuval-Davis (1997), it is no accident that those who are preoccupied with the racial “purity” of the nation should also be preoccupied with the sexual relations between members of different collectives. Just as the denigration of the racialised Other cannot be separated from the process of constructing the white cisgendered “self,” the construction of the white heteronormative family cannot be separated from the nativist project of border control. The premise of separating “new” and “classic” gender issues in radical right studies, focused on operations of the state and within the home, respectively, upholds the public/private boundary that has been widely disregarded in feminist literature as inadequate for analyses of the construction of civil society (Chatterjee, 1990). This appears to be another site in radical right scholarship upholding the very formations it seeks to analyse, preventing itself from engaging with the intersectional form of the racism and sexism within radical right ideology.

Scrinzi (2024) describes “ethnicity regimes” as intrinsically gendered: “different histories of colonialism and immigration and different configurations of ethnic majority/minority relations shape specific repertoires of racialisation and whiteness, which variously incorporate models of gender and sexuality,” (p. 10). Analogously, Butler (2024) points to the colonial history of gender dimorphism, or the idealisation of the obligatory gender binary as a heteronormative, white, and European norm, suggesting a “metonymic link” between gender and race. Academic debate over whether or how “central” gender should be considered to the radical right's ideology misapprehend the nature of intersectionality. As Connell (2005) reminds us, gender permeates the social world at every level, regardless of our unit of analysis. To claim that gender does not define the radical right is erroneous—but so too would be the notion that the radical right is uniquely gendered in comparison to other party families. We find gender everywhere we look. The distinction we are making therefore should not be in reference to degree—of “how central” gender is to the ideology of the radical right, but in difference of kind.

### 5.3 Variation within and variation between

It seems important here to address the point raised by Scrinzi (2024), that clear variations exist diachronically and synchronically across radical right parties. She suggests that the policy and discourse of the radical right is not “monolithically sexist” but is complex, arising from strategies to attract support, to modernise public image, and from variation of respective historical contexts. Some parties engage in strategic practises of homonationalism, while others such as Poland's PiS (Graff and Korolczuk, 2022) or Hungary's Fidezs retain overtly homophobic rhetoric. Similarly, PiS explicitly targets feminism as “un-Polish” (Graff and Korolczuk, 2022), whereas Off (2023) notes the Alternative für Deutschland's endorsement of “second wave” feminist ideas—attributing this to the party's post-socialist context—though they firmly reject gender as a social construct. The same tension between difference of degree or kind rears its head. As argued in previous sections, the increased participation of women within the radical right, or instrumentalisation of feminist language does not detract from the sexism or anti-feminism of these parties. Nor does choice in euphemism to uphold racial exclusion detract from racism, or employment of sexual politics necessitate contribution to queer liberation.

Accounting for the variation between radical right parties is useful if the “radical right” is our class of comparison. If we are conceptually defining these parties as a “family” in relation to others, the above discussion outlines how this entails looking to the deep and broad continuities these parties share. Freeden (1998) considers ideology to be a stable phenomenon, expressing doubt as to whether neatly bounded “ideologies” can be assigned to individual parties on a one-to-one basis. I would argue that the problem posed by Scrinzi (2024) has less to do with a fragmentation of “ideology” between individual radical right parties, and more to do with conceptually looser constructs, such as “framings” which account for the discursive work performed by political actors, shaped by political-institutional and cultural-discursive opportunities open to them (Caiani and Della Porta, 2011). Despite variation in these framings, these parties remain ideologically unified in refusal to interrogate or dismantle foundational systems of heteronormativity, cisnormativity, patriarchy, and racism. Endorsement of select progressive values in these instances serve exclusionary ends, not ideological evolution (Off, 2023).

A final tension that arises here is that of exceptionalism: how do we distinguish the racialised sexist ideological architecture of the radical right while accounting for that which is also prevalent within the mainstream establishment? I might offer here that the establishment upholds white supremacy and male dominance through the ideological veneers of liberalism, meritocracy, and equality of opportunity. They form precisely the terrain that Haslanger (2017) urges us to critique: oppressions that persist not through explicit rejection of equality, but through the co-option of its language. I would also propose that meta-politically, the radical right provides multiple important functions for the establishment—as a “character foil” by which it can distinguish itself as “not” what the radical right so explicitly *is*, pointing with horror at such overt displays of racialised sexism. Also, as outlined by Valentim (2024), the radical right paves the way to normalising policy that the establishment can shift towards when it proves electorally useful, highlighting the flimsiness of liberal dedication to “equality”.

The ideology of the radical right is one coloured by what Connell (2005) would refer to as a heightened gender-consciousness, animated by anxiety—of status loss, of feminisation, of changes to racial hierarchies. This project is not simply defined by conservatism, but by a reactionary, nostalgic intensity that distinguishes it from quieter, more adaptable modes of dominance embedded in mainstream political entities. White masculinity specifically contributes to this intensity through its historical status as the normative subject, which grants it power in terms of political effectiveness. When this normativity is destabilised, through critique of feminist or critical race scholarship, or the increased visibility of alternative identities and ways of living, the result is a perception not merely of loss, but of a disruption to a naturalised sense of authority and belonging. On this note however, much scholarship has articulated this ideology as one that is explicitly “masculine” (Ralph-Morrow, 2022). In keeping with earlier discussion of this paper, we must ask: what does this mean? Is “masculinity” being used synonymously with “conservatism” or “patriarchy”? We need to move beyond the indiscriminate term of “masculine” towards more scientifically specific concepts to discuss how the political phenomenon advances a particular articulation of “masculinity”—or rather, of racialised gender relations more broadly.

Academically, we can offer that these differences between the liberal mainstream and of the radical right are of species rather than of degree. Nonetheless, the dramatic shift rightwards of the mainstream establishment across Europe, concomitant with the success of the radical right, and the outcomes this will have on migrants as racialised figures, on trans folk, on women, on any individual who stands in opposition to the cis-heteronormative white norm of Europe, calls into question whether this “conceptual specification” is one worth making.

## 6 Power relations

According to Stenger and True (2024), “gendered power dynamics pertain to the hierarchical order of masculinities and femininities… vis-à-vis the state and/or global environment that is prefigured into their ideologies,” (p. 8). In this section, I examine the concept of hegemonic masculinity as a central organising principle of gendered power relations, critiquing how the concept is often applied in radical right studies, and further returning to the inherent tension between literature attempting to account for the “dominant subjectivities” of the radical right, vs. that which is trying to account for social class, and the intimate relationship between masculinity, technology, and capitalism.

### 6.1 Hegemonic masculinity

Hegemonic masculinity, derived from the original Gramscian concept and stripped of its class underpinnings, describes the “configuration of gender practise which embodies the currently accepted answer to the problem of the legitimacy of patriarchy, which guarantees (or is taken to guarantee) the dominant position of men and the subordination of women,” (Connell, 2005: 77). The concept of “hegemonic whiteness” (Hughey, 2010) has also been developed to account for the cultural process of white identity formation based on the reproduction of racist ideology, though Christensen and Jensen (2014) consider whiteness a key component of hegemonic masculinity itself. Many have critiqued the uneasy position of the concept in the context of Connell's larger body of work, regarding the difficulty in reconciling the “ideal” quality of hegemonic masculinity with Connell's insistence that all masculinities are configurations of practise (Garlick, 2016; Schippers, 2007). Beasley (2008) points to pervasive “slippages” in its usage, as a political mechanism, as a descriptor of dominant forms of masculinity, and as a referent to actual groups of men.

It is my contention that such “slippages” characterise the use of the concept in radical right scholarship: Dietze and Roth (2022) refer to radical right actors as “structurally hegemonic speakers”: Miller-Idriss and Pilkington (2017) assert that far-right cultures reflect “aspects of hegemonic masculinity”; Sauer (2020) emphasises the place of hegemonic masculinity in right-wing narratives and imagination. Certainly, when we consider some of the “hegemonic principles” upon which the concept is stabilised such as heterosexuality and whiteness, the ideology of the radical right aligns well. Nonetheless, it is difficult to speak of this discourse as imbued with more fundamental bases crucial to hegemony, such as legitimacy, authority, and consent. While the radical right exists within global and national contexts shaped by hegemonic masculinity, framing the party family as an agent of its proliferation is a misapplication of the concept. Adjacent to previous discussion on the ubiquity of “masculinity” in this literature, it appears that scholars are reliant on an impression the concept freely loans in relation to unequal power dynamics, often conflating it with that which is “conservative” or “patriarchal.” While its use is not meaningless *per se*, treating the concept as interchangeable with broad ideological commitments of a party family flattens it as a theoretical tool.

Other more nuanced analyses integrate concepts such as “dialectical pragmatism” (Demetriou, 2001) and “hybrid masculinities” (Bridges and Pascoe, 2014) to account for the pervasive claims to victimhood made by men of the far-right at-large, whereby hegemonic masculinity is framed as a strategy, borrowing aspects of other subordinate masculinities that are useful for continued domination (Ging, 2019). Such analysis, attending to the pervasive claims to victimhood of men that occupy dominant social categories, still stop short of confronting the deeper conceptual problems that lie unresolved at the heart of hegemonic masculinity. As argued by Garlick (2016), hegemonic masculinity is not something that takes a singular form or can ever be fully achieved or embodied. Moreover, I believe this research faces a more fundamental issue in the recurrent uneasy integration of “class” into its analysis.

Earlier in this paper, I critiqued “class-only” approaches for their neglect of dominant subjectivities—those of white European men who remain overrepresented in the occupational groups that form key constituencies of radical right support. Conversely, this literature integrating masculinity theory into radical right scholarship appears dedicated to the reaffirmation of this dominance, disregarding the role of class as its effects cannot be squared neatly in terms of economic inequality, and thus more abstractly in relation to “injustice” (Fraser, 1995). White masculinity is not a monolith: it is internally differentiated both within and beyond the radical right—class plays an unignorable role in this differentiation. The vast majority of white men do not find political resonance with radical right parties, underscoring the need for class to be taken seriously as a relevant force in structuring which men do, even if it is not singularly determinative. Within the radical right, as previously mentioned, Damhuis (2020) reveals the heterogeneity in socio-structural background of these voters, in contrast with earlier models by those such as Oesch and Rennwald (2018) and Kurer (2020) who's work emphasises ideal-typical voter classifications. This suggests the need for frameworks capable of accommodating both internal and external inflections and fragmentations of white masculinity along class lines.

### 6.2 Capitalism, technology, and masculinity

According to Garlick (2020), “masculinity” in the West has been defined predominantly in relation to paid work. Indeed, there exists a rich body of historical, anthropological, and sociological scholarship, generally under the banner of “deindustrialisation studies” that have chronicled the social deconstruction and transfiguration of gender relations following the post-industrial transition, revealing the central role of work as a site of the construction and means of expression of white masculinity (Wight, 1994; Walkerdine and Jimenez, 2012; McIvor, 2013). Cornwall et al. (2016) note that men often fail to reconcile normative masculinities with new economic realities and are left struggling to realise masculine ideals that run counter to their actual material conditions of existence.

The development of capitalism has led to a shift in who Salzinger (2016) calls the “privileged subject of exploitation,” from the putatively breadwinning patriarch of the global North, to precariously earning women of the global South, as capital has been released from the constraints of Fordism. The unignorable role of capital and technology complicates any clear binary between who is the “objective” and who is the “phantasmatic” victim in the era of neoliberal restructuring (Zalloua, 2024). The white men who disproportionately make up the radical right electorate for example, while heterogenous in their socio-structural backgrounds, are certainly not a concentration of economic “elites.” Are the forms of exploitation experienced by women of the global South resulting from accelerated globalisation qualitatively “worse” than that of the dislocated blue-collar worker of the global North? Undoubtedly. But is ranking forms of exploitation into a dualism of better/worse, or oppressed/unoppressed what we are after, analytically speaking?

I would argue that a difficulty in stepping away from these binaries heavily characterises both “class-centric” and more heavily gendered inquiry into the radical right. The aim of any intersectional approach should not be the swapping-out of one totalising explanation with another: we should remain as suspicious of the simplicity of “threatened status” explanations of radical right support as we are of those pertaining to “relative economic deprivation.” This does not necessitate debating the dominance of the men who are the analytic subjects of this research—rather, I want to question the premise of these binaries. Their utility appears contingent on analytical endeavour: if our aim is to foreground marginalised identities and articulate structures of exclusion, the distinction between dominance and oppression is not only appropriate but necessary. When our focus is on understanding the radical right itself—its appeal, ideological architecture, socio-structural origins—sole reliance on the category of “dominant” risks flattening important complexities whose existence we should not deny for the sake of analytic neatness. Capitalist transformation in the post-industrial era again shifts the material base upon which masculine prerogatives rest (Schwarzkopf, 2014). We should not necessarily have to capitulate these shifts into frameworks of “objective” economic inequality, or of discursive claims to victimhood, to acknowledge the analytic utility in examining that they alter something profound in the way that dominance is *felt*, destabilising access to channels by which it can be performed, and in many cases, leading to attempts to find new (political) avenues through which it can be reconstituted. This interaction between capital and subjectivity highlights yet another way that masculinity is conceptualised—as not just restricted to the symbolic realm, as social practises are always already entwined with the symbolic in constituting and reproducing masculinities as material-discursive entities (Garlick, 2016).

## 7 Conclusion

Labelling an analysis as “intersectional” is intimidating—there will invariably be social categories that are not adequately accounted for. I have intended its use here as a point of departure, not of arrival, the possibilities for its application to the study of the radical right remains open-ended. The intention of this paper has been to combine macro-sociological understandings of racialised gendered power relations with micro-functional analyses of white men's identities and behaviours. In doing so, I have attempted to elaborate on the idea of “superordinate intersectionality” to examine social categories that intersect to premise normative positions of dominance. Here, I have argued for the re-working of the use of the concept of “masculinity” as it is currently employed in radical right literature, to describe both the identity and the ideology of these parties. I have argued that these identities are not fully accounted for if we do not take into consideration whiteness, and social class. Similarly, I have argued that the defining ideology of “nativism” underpinning much of what distinguishes radical right as a party family is a fundamentally racialised and gendered construct.

This paper ends with a call for approaches that can reconcile consideration of dominant subjectivities with material transformation under capitalism, thus brushing up against more fundamental issues in the social and political sciences of materialist vs. post-structuralist approaches. We must attempt to resist the reproduction of further binaries that are so persistent in Western thought (Haraway, 1991), and thus in radical right ideology too: self/other, culture/nature, male/female, civilised/primitive. Reducing our subjects to solely “dominant” overdetermines them inasmuch would reducing them to “oppressed.” Racism is reductive, sexism is reductive, fascism and authoritarianism are reductive. Our explanations for how these political phenomena come about need not be. Hopefully, this discussion illuminates further the need for an intersectional approach, one that neither denies the role of capitalist development as intimately entangled with racialised gendered subjectivities, nor accepts at face value the narratives of loss and victimhood it has mobilised.
